# Support-Seeking After Market Withdrawal of a Purpose-Built AI Well-Being Tool (Ash): Cross-Sectional Survey

**DOI:** 10.2196/96515

**Published:** 2026-10-13

**Authors:** Caitlin A Stamatis, Kaylee P Kruzan, Josh Hsu, Mark A Ungless, Thomas D Hull

**Affiliations:** 1 Slingshot AI New York, NY United States; 2 Feinberg School of Medicine Northwestern University Chicago, IL United States

**Keywords:** AI, LLM, mental health, regulation, policy, digital health, large language model, artificial intelligence

## Abstract

**Background:**

Purpose-built AI well-being tools—designed with clinical safeguards for everyday emotional difficulties such as stress, grief, and relationship conflict—represent a growing category of digital mental health support. When such tools are removed from the market, users must navigate alternatives on their own; however, no empirical data exist on where they actually turn.

**Objective:**

We aimed to characterize real-world support-seeking after the withdrawal of a purpose-built AI well-being mobile app (Ash), specifically to (1) document where former users turned for emotional and mental health support, (2) characterize how general-purpose AI tools were used for mental health, (3) assess perceived changes in service access and subjective well-being, and (4) capture qualitative experiences of the transition.

**Methods:**

We conducted a cross-sectional survey of 393 UK-based adults who had used Ash within 90 days before its withdrawal from the UK market in January 2026. Of 7415 randomly sampled eligible users invited by email, 393 (response rate 5.3%) responded and 364 (92.6%) completed all items. The survey assessed self-reported sources of emotional and mental health support following withdrawal, use of general-purpose AI tools, perceived changes in access to support, and subjective well-being. Free-text responses (n=272) were analyzed using codebook-based qualitative content analysis, with the codebook developed through a hybrid inductive-deductive process. Reporting follows the CHERRIES (Checklist for Reporting Results of Internet E-Surveys) guidelines.

**Results:**

The most common primary sources of mental health support since losing access to a purpose-built AI well-being application were general-purpose AI tools (27.1%, 104/384; 95% CI 22.9-31.7) and no support at all (26%, 100/384; 95% CI 21.9-30.7). Relatively fewer respondents reported turning to National Health Service services (19/384, 4.9%; 95% CI 3.2-7.6) or a private therapist (8.1%, 31/384; 95% CI 5.7-11.2) as their primary resource. Among the 157 respondents reporting use of general-purpose AI for mental health support, the dominant motivations were instant access (78.3%, 123/157) and cost (73.9%, 116/157) rather than perceived helpfulness (18.5%, 29/157). Overall, 60.2% (219/364; 95% CI 55.1-65.1) reported worse access to support, rising to 80.7% (71/88; 95% CI 71.2-87.6) among those who had not sought support since. Qualitative themes converged on these patterns: 33.8% (92/272) reported finding no suitable alternative, 22.8% (62/272) judged general-purpose AI inferior, and 15.8% (43/272) expressed emotional distress at the loss.

**Conclusions:**

In this exploratory, retrospective survey, removing a purpose-built AI well-being tool was not associated with a redirection to human-led mental health services; most respondents turned to general-purpose AI or stopped seeking support entirely, and those who disengaged reported the poorest well-being. Regulatory frameworks should account for real-world care-seeking behavior when evaluating the risks and benefits of purpose-built AI tools for general wellness.

## Introduction

As demand for mental health support far exceeds available supply [1], a growing number of people are turning to generative AI for emotional support [2,3]. A recent national survey found that 13% of US adolescents and young adults have used generative AI for mental health advice, with rates exceeding 22% among those aged 18 to 21 years [2]. A systematic review of 79 studies documented the rapid expansion of generative AI applications across mental health diagnosis, therapy, and clinician support [3]. The safety profile of many AI tools, however, remains concerning: studies have found that general-purpose AI chatbots frequently fail to recognize or appropriately respond to mental health crises [4], and even in nonacute settings, emerging evidence links their use to emotional dependency and reinforcement of harmful beliefs [4-7]. This creates a tension at the heart of digital mental health policy: millions of people are already using general-purpose AI tools for emotional support, while purpose-built AI tools designed for safe, effective psychological support face the highest regulatory barriers. What remains poorly understood is what happens when users who have access to a purpose-built AI well-being tool, designed with clinical safeguards and psychological expertise, lose access to that tool and are left to navigate the broader mental health support landscape on their own.

The gap between demand for mental health support and available supply is a global challenge, and one that has underscored a need to consider different support channels for the wide range of presentations that may drive help-seeking behavior. Even in high-income countries with well-resourced health systems, up to two-thirds of patients with depression receive no treatment [8] and wait times for psychological services routinely stretch to months or years. In the United Kingdom (the setting for the present study), an estimated 1.8 million people are currently on National Health Service (NHS) waiting lists for mental health services [9]. Mental health costs the UK economy an estimated £118 billion per year [10]. These figures span a broad spectrum from clinical disorders requiring specialist intervention to the everyday stress, relational difficulties, and life transitions that characterize most help-seeking. The UK Government has explicitly recognized this distinction: the Department of Health and Social Care’s 2022 Mental Health and Wellbeing Plan states that “poor wellbeing is distinct from mental illness, and we do not want to ‘medicalise’ the normal worries and stresses of life” [11]. Social prescribing, the practice of referring patients to nonclinical community services, now embedded in primary care across the United Kingdom, reflects the same principle: not all support for emotional well-being requires clinical intervention [12].

Against this backdrop, a new category of digital tool has emerged: AI well-being applications designed specifically for the everyday emotional difficulties (eg, stress, grief, relational conflict, or life transitions) that constitute much of the unmet demand described in the preceding paragraph. These users may occupy what has been termed the “missing middle” of mental health care: people whose needs exceed self-help but who do not meet thresholds for specialist services [13]. Critically, the sustainability of such tools is not guaranteed, particularly in light of evolving regulatory policy. The NASSS framework (Non-adoption, Abandonment, Scale-up, Spread, and Sustainability) highlights how regulatory, organizational, and individual-level complexity can interact to disrupt access to health technologies, particularly when disruption originates outside the user-technology relationship [14]. What happens to users caught in such a disruption, however, has not been studied.

Purpose-built AI well-being tools extend the principle that not all emotional support requires clinical intervention into the digital realm, offering accessible, individualized support for stress management, resilience-building, and relational difficulties without requiring clinical resources or diagnostic labeling [15]. However, global regulatory frameworks governing such tools have not kept pace with either the technology or the demand. In the United States, the Food and Drug Administration’s General Wellness Policy provides a structured pathway for low-risk wellness products that do not make diagnostic or treatment claims [16], and the Food and Drug Administration has adopted a policy of enforcement discretion for certain lower-risk digital health software functions [17]. The European Union’s Medical Device Regulation explicitly states that “software intended for life-style and well-being purposes is not a medical device” [18]. In the United Kingdom, the Medicines and Healthcare Products Regulatory Agency has developed regulatory guidance for Digital Mental Health Devices, but the distinction between medical devices and well-being products is challenging to navigate; while, as of July 2026, the Medicines and Healthcare Products Regulatory Agency has created a regulatory sandbox that remains in testing [19], there is not yet a dedicated regulatory pathway for AI well-being products that clearly distinguishes them from medical devices. In practice, this means that purpose-built well-being tools may face medical device classification while general-purpose AI platforms, which are used at far greater scale for the same purposes and without equivalent safeguards, remain unregulated for mental health purposes in the United Kingdom. We use the term “unregulated” throughout the paper to mean “not regulated as a medical device for mental health purposes.”

This regulatory asymmetry has real-world consequences. Ash, a consumer well-being mobile app developed by Slingshot AI for adults managing everyday emotional difficulties, was voluntarily withdrawn from the UK market in January 2026 following regulatory uncertainty about a general wellness pathway in the United Kingdom. The app was built alongside clinical psychologists with clinically informed safeguards and had a growing evidence base, including multiple single-arm trials showing improvements in loneliness, perceived social support, and social engagement [20,21], and an analysis of over 20,000 real-world conversations demonstrating that purpose-built suicide risk safeguards operated as designed [22]. The United Kingdom withdrawal thus defined a population of active users and temporal context for this post-withdrawal descriptive survey study.

The present study leverages this voluntary market withdrawal to address a question with implications beyond any single product or regulatory decision: when users of a purpose-built AI well-being tool lose access, where do they actually go? Specifically, we aimed to (1) document where former users turned for emotional and mental health support, (2) characterize how general-purpose AI tools are being used for mental health in this population, (3) assess perceived changes in access to support and subjective well-being, and (4) capture qualitative experiences of the transition through open-ended responses. These aims correspond to four research questions (RQs): (RQ1) Where do former users turn for mental health support after losing access to a purpose-built AI well-being tool? (RQ2) How are general-purpose AI tools used for mental health support in this population? (RQ3) How do perceived access to support and subjective well-being change following service disruption? (RQ4) How do former users describe their experience of the transition?

## Methods

### Study Design and Context

This cross-sectional survey study examined where users of a purpose-built AI well-being mobile app turned for mental health support following an involuntary service disruption. The voluntary withdrawal of Ash from the UK market in January 2026, described above, created a defined study population and clear temporal boundary for the disruption. This study is described in accordance with the CHERRIES (Checklist for Reporting Results of Internet E-Surveys; Multimedia Appendix 1) [23].

### Product Context

Ash is an AI specifically designed to support mental health. Purpose-built generative AI tools for mental health are distinct from general-purpose AI in that, at a minimum: (1) the base model distribution is modified through pretraining and fine-tuning to address mental health concerns, (2) the system architecture is further aligned through mental health-specific guardrails and targeted predeployment (“offline”) evaluation procedures, and (3) reinforcement learning is driven by reward signals focused on health outcomes and clinical appropriateness in addition to or instead of pure engagement or user satisfaction signals [20,22,24]. For additional detail on the Ash model and product development, see [20,21].

### Participants and Recruitment

Eligible participants were UK-based adults (aged 18+ years) who had used Ash (which was available for free at the time) within the 90 days before its withdrawal; no further inclusion or exclusion criteria were applied. Invitations were sent by the Slingshot AI team to the email address registered to each user’s Ash account, with 7415 invitees drawn at random from all eligible users in the database. The survey was distributed through Tally [25] between February and March 2026. Of the 7415 invited, 618 (8.3%) opened the survey, of whom 393 (63.6%) responded, yielding an overall response rate of 5.3% (393/7415). Two follow-up reminders were sent by email to those who did not respond. A total of 364 (92.6%) completed all items; the remaining 29 respondents responded to the first half of the survey (support sources, AI use patterns) but did not complete the final section (perceived access change, well-being, and demographics). All 393 respondents were included in analyses where they provided data; survey responses were deidentified before analysis. Participants were provided the option of entering a prize draw alongside the survey.

### Ethical Considerations

This study was reviewed in March 2026 by the BRANY (Biomedical Research Alliance of New York) independent Institutional Review Board and determined to be exempt under category 4(ii) (File # 26-081-2393). These data were collected during routine product operations from users who had consented to the use of their data for research, and the deidentified dataset was analyzed under this exemption. The survey invitation described an anonymous, voluntary survey whose aggregate results would inform product and policy work (see email invitation text in Multimedia Appendix 2). As this study involved community-based UK adults rather than NHS patients or records, it fell outside the UK Health Research Authority’s NHS Research Ethics Committee system. All data processing complied with UK General Data Protection Regulation and the Data Protection Act 2018.

### Measures

#### Overview

The survey comprised 12 items assessing five domains: (1) support sources since losing access to Ash, (2) general-purpose AI use patterns, (3) perceived change in access to support, (4) subjective well-being, and (5) demographics. Items were developed by the research team to capture the specific phenomenon of interest, namely, where users turn when a purpose-built mental health AI tool becomes unavailable.

As the survey comprised single-item and multiple-response measures of behavior and self-perception rather than multi-item reflective scales of latent constructs, internal-consistency reliability (eg, Cronbach α) and discriminant-validity indices were not calculable; each item was intended as a direct indicator of the behavior or experience it names. The full instrument, including all item stems and response options, is provided in Multimedia Appendix 2.

#### Support Sources

Respondents were first asked, “Since Ash/Slingshot stopped being available in the United Kingdom, where have you turned for emotional or mental health support?” Respondents could select all that apply from 11 options: general-purpose AI tools (eg, ChatGPT [OpenAI], Claude [Anthropic], Gemini [Google LLC], and Copilot [Microsoft Corp]); other mental health or well-being apps; NHS services (general practitioner, NHS Talking Therapies, etc); private therapist/counsellor; friends, family, or partner; support groups or community groups; workplace support/employee assistance program; online forums or social media; books, podcasts, or self-help programs; “I haven’t sought support since”; and other (free text). A follow-up item asked respondents to identify which single source they had relied on most.

#### General-Purpose AI Use Patterns

Four items assessed AI use among all respondents. Frequency of use for emotional or mental health support was assessed on a 7-point scale (daily to never). For those endorsing general-purpose AI use for emotional or mental health support, 3 follow-up questions were asked: (1) The role in the respondent’s life, assessed on a 5-point scale (main source of support to occasional curiosity); (2) primary uses of AI for mental health, assessed via a multiselect item (“select up to 3”) with options including venting or feeling heard; advice or problem-solving; coping tools; relationship or communication help; stress/anxiety/sleep support; and journaling or reflection prompts; (3) motivations for choosing AI, assessed via a separate multiselect item with options including free or low cost, instant access, felt more helpful than alternatives, more private, easier than NHS or human support, didn’t want something “medical,” and “other” options.

#### Perceived Access Change

Respondents rated how their access to mental health support had changed since losing access to Ash on a 5-point scale (much better to much worse).

#### Well-Being

A single item assessed general well-being over the past month on a 5-point scale (much better than usual to much worse than usual).

#### Open-Ended Response

One free-text item asked, “Is there anything you’re using now that works better—or worse—than Ash did for you?”

#### Demographics

Age range (6 categories from 18-24 to 65+ years) and UK region (England, Scotland, Wales, Northern Ireland, and prefer not to say).

### Data Analysis

#### Quantitative Analysis

Descriptive statistics (frequencies and percentages) were computed for all structured items. For multiselect items, percentages reflect the proportion of all respondents who endorsed each option. Denominators vary slightly across items due to partial completion and are reported where relevant. Analyses were done in Python 3.10 using pandas. All proportions are reported to one decimal place as percentages with numerators and denominators, accompanied by Wilson score 95% CIs; qualitative code frequencies are reported as counts and percentages of the 272 analyzable free-text responses. Consistent with this study’s descriptive aims, no null-hypothesis significance testing was conducted.

#### Qualitative Analysis

Free-text responses were analyzed using codebook-based qualitative content analysis, with the codebook developed through a hybrid inductive-deductive process [26]. Of 351 free-text responses, 272 contained sufficient content for analysis (>5 characters, excluding single-word or uninformative entries). The lead author, a licensed clinical psychologist, first read all responses and inductively identified recurring patterns, which were discussed and refined with the authorship team and organized into a codebook of 15 nonmutually exclusive codes across five domains: (1) evaluation of general-purpose AI as a substitute, (2) support gap and disengagement, (3) emotional response to loss, (4) valued features of a purpose-built tool, and (5) coping strategies adopted. The full codebook, including code definitions, inclusion and exclusion rules, and anchor examples for different codes, is provided in Multimedia Appendix 3. Each code was then operationalized as a set of transparent keyword and phrase patterns (regular expressions). Anthropic’s Opus 4.6 was used to assist in drafting candidate patterns from the code definitions; all patterns were reviewed, edited, and approved by the lead author, and the classification step itself was fully deterministic (rule-based rather than model-based), such that identical inputs yield identical codes; no generative model was involved in assigning codes to responses. Patterns were refined over 3 coding passes. After each pass, the lead author manually coded those that remained uncoded, also reviewing all responses to correct misclassifications, either by refining the shared patterns or by direct reclassification. The 24 direct reclassifications are individually documented, with rationale, in this study’s audit records and summarized in Multimedia Appendix 3. The final coded dataset was reviewed by the full authorship team for face validity. Of the 272 responses, 270 (99.3%) were coded to at least one theme; the remaining 2 lacked sufficient specificity for classification and are described in Multimedia Appendix 3. Coding was performed by a single coder, and interrater reliability statistics were therefore not computed. Representative quotations were selected to illustrate each theme. The codebook, deterministic classification rules, and a summary of every manual reclassification are publicly available; because the free-text responses are not publicly shared to protect participant privacy, response-level reproduction is limited by data availability.

#### Reflexivity and Coder Roles

Coding was led by the first author, a licensed clinical psychologist with research expertise in digital mental health employed by Slingshot AI, the developer of Ash, at the time of the analysis; 1 coauthor (KPK) is an academic researcher unaffiliated with the company. The authorship team recognizes that our proximity to the tool under study could shape codebook construction and interpretation, particularly given that respondents were former users of a withdrawn product; however, we attempted to mitigate this risk in part by including an independent academic author who provided input into the iterative development of the codebook and coding decisions. Three additional features of the design help mitigate risk of bias: (1) the codebook includes codes capturing unfavorable evaluations of Ash and favorable evaluations of general-purpose alternatives, both of which are reported in full; (2) code assignment was deterministic rather than interpretive; and (3) all coding decisions are independently reproducible from the shared analysis materials.

## Results

### Sample Characteristics

Three hundred ninety-three former Ash users responded to the survey (response rate: 5.3%, 393/7415). Of the 363 (92.4%) users providing demographic data, 30.3% (110/363) were aged 25-34 years, 25.6% (93/363) were 35-44 years, and 16.5% (60/363) were 45-54 years, with 12.1% (44/363) aged 18-24 years and 14.9% (54/363) aged 55 years or older. The regional distribution was broadly representative of the UK population, with 87.3% (317/363) of respondents based in England, 6.3% (23/363) in Scotland, 4.4% (16/363) in Wales, and 0.6% (2/363) in Northern Ireland (Table 1). Respondents were generally more engaged users (median of 10 sessions, IQR 4-27 sessions) than nonrespondents (median of 2 sessions, IQR 1-6 sessions); the geographic distribution (ie, UK region) of users did not significantly differ between respondents and nonrespondents.

**Table 1:** Sample demographics (n=363). Demographics were completed by 363 of 393 (92.4%) respondents.

Characteristic		Values		
**Age range (years), n (%)**				
	18-24	44 (12.1)		
	25-34	110 (30.3)		
	35-44	93 (25.6)		
	45-54	60 (16.5)		
	55-64	41 (11.3)		
	65+	13 (3.6)		
	Prefer not to say	2 (0.6)		
**UK region, n (%)**				
	England		317 (87.3)	
	Scotland		23 (6.3)	
	Wales		16 (4.4)	
	Northern Ireland		2 (0.6)	
	Prefer not to say		5 (1.4)	

### Support Sources Following Service Withdrawal

Table 2 presents the sources of support respondents reported turning to since Ash became unavailable (n=389 answering this multiselect item). General-purpose AI tools (eg, ChatGPT, Claude, and Gemini) were the most endorsed option, selected by 40.4% (157/389) of respondents (95% CI 35.6-45.3). This was followed by friends, family, or partner (131/389, 33.7%; 95% CI 29.2-38.5) and “I haven’t sought support since” (117/389, 30.1%; 95% CI 25.7-34.8). Notably, only 16.5% (64/389; 95% CI 13.1-20.5) reported turning to a private therapist or counselor, and 14.9% (58/389; 95% CI 11.7-18.8) to NHS services.

**Table 2:** Support sources since Ash became unavailable. “Selected (multi)” reflects all-that-apply responses (n=389 answering). “Primary” reflects the single source relied on most (n=384 answering); percentages use each column’s denominator.

Support source	Selected (multi, n=389), n (%)	Primary (n=384), n (%)
General-purpose AI tools	157 (40.4)	104 (27.1)
Friends, family, or partner	131 (33.7)	63 (16.4)
“I haven’t sought support since”	117 (30.1)	100 (26)
Other mental health or well-being apps	69 (17.7)	21 (5.5)
Private therapist or counselor	64 (16.5)	31 (8.1)
Books, podcasts, or self-help	62 (15.9)	11 (2.9)
NHS^a^ services	58 (14.9)	19 (4.9)
Online forums or social media	33 (8.5)	7 (1.8)
Support groups	21 (5.4)	8 (2.1)
Workplace support or EAP^b^	12 (3.1)	0 (0)
Other	27 (6.9)	17 (4.4)

^a^NHS: National Health Service.

^b^EAP: employee assistance program.

When asked to identify their single most relied-upon source (Figure 1), general-purpose AI tools remained the most common response (27.1%, 104/384; 95% CI 22.9-31.7), followed closely by “I haven’t sought support since” (26%, 100/384; 95% CI 21.9-30.7; note that 4.9% (19/389) of participants selected “I haven’t sought support since” alongside other response options) and friends, family, or partner (16.4%, 63/384; 95% CI 13.0-20.4). Fewer respondents reported using NHS services (4.9%, 19/384; 95% CI 3.2-7.6) or a private therapist (8.1%, 31/384; 95% CI 5.7-11.2) as their primary resource.

**Figure 1:**
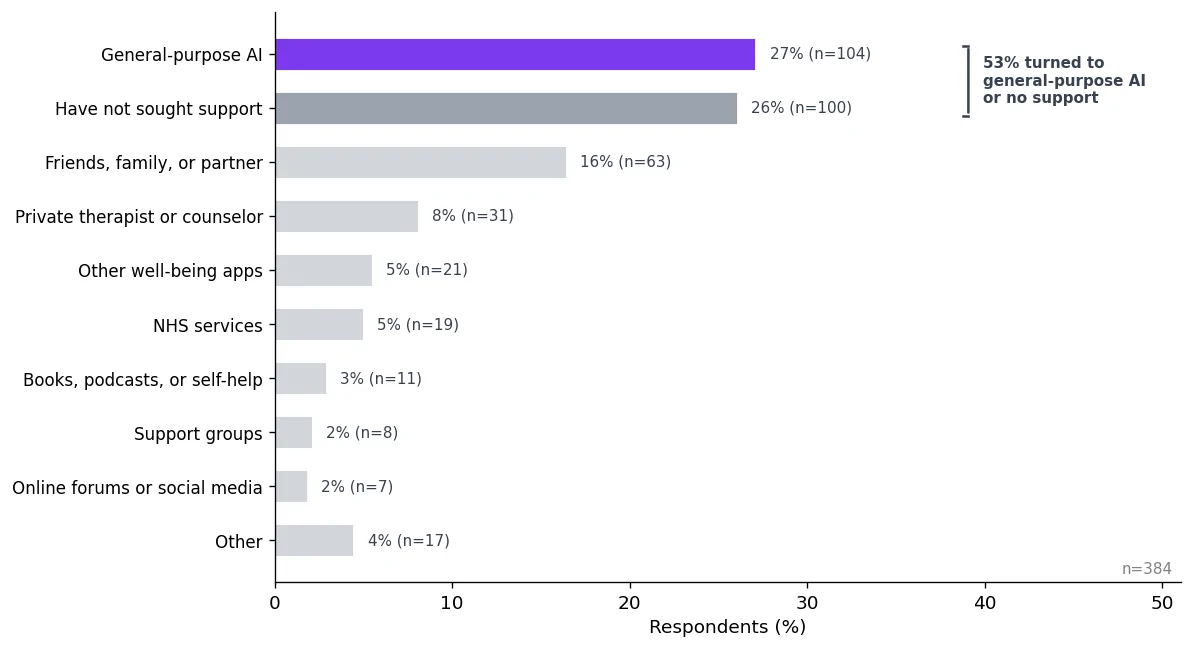
Primary source of support since Ash became unavailable (n=384). Purple bar indicates general-purpose AI tools and the dark gray bar indicates respondents who have not sought support. NHS: National Health Service.

### General-Purpose AI Use Patterns

In the subset of participants who identified general-purpose AI as one of their sources of mental health support (n=157), 154 responded to a question asking about their usage patterns. Use was frequent: 78.6% (121/154; 95% CI 71.4-84.3) used it weekly or more, with 24.7% (38/154; 95% CI 18.5-32.0) reporting daily use. In terms of how AI mental health support fits into their lives (n=152 responding), 31.6% (48/152; 95% CI 24.7-39.3) described it as their main source of support, 43.4% (66/152; 95% CI 35.8-51.4) as one of several sources, 14.5% (22/152; 95% CI 9.8-20.9) as a backup when nothing else was available, and 9.9% (15/152; 95% CI 6.1-15.6) used it occasionally or out of curiosity. The most commonly reported use cases (multiselect; percentages of all 157) were advice or problem-solving (77.1%, 121/157; 95% CI 69.9-83.0), venting or feeling heard (54.8%, 86/157; 95% CI 47.0-62.4), coping tools such as grounding or cognitive reframing (36.9%, 58/157; 95% CI 29.8-44.7), relationship or communication help (35%, 55/157; 95% CI 28.0-42.8), and stress, anxiety, or sleep support (22.9%, 36/157; 95% CI 17.0-30.1). The primary motivations for choosing general-purpose AI (Figure 2) were instant access (78.3%, 123/157; 95% CI 71.3-84.1) and cost (73.9%, 116/157; 95% CI 66.5-80.1), followed by ease relative to NHS or human support (33.1%, 52/157; 95% CI 26.2-40.8), privacy (19.1%, 30/157; 95% CI 13.7-26.0), and perceived helpfulness compared to alternatives (18.5%, 29/157; 95% CI 13.2-25.3). Only 7% (11/157; 95% CI 4.0-12.1) cited not wanting something “medical” as a motivation.

**Figure 2:**
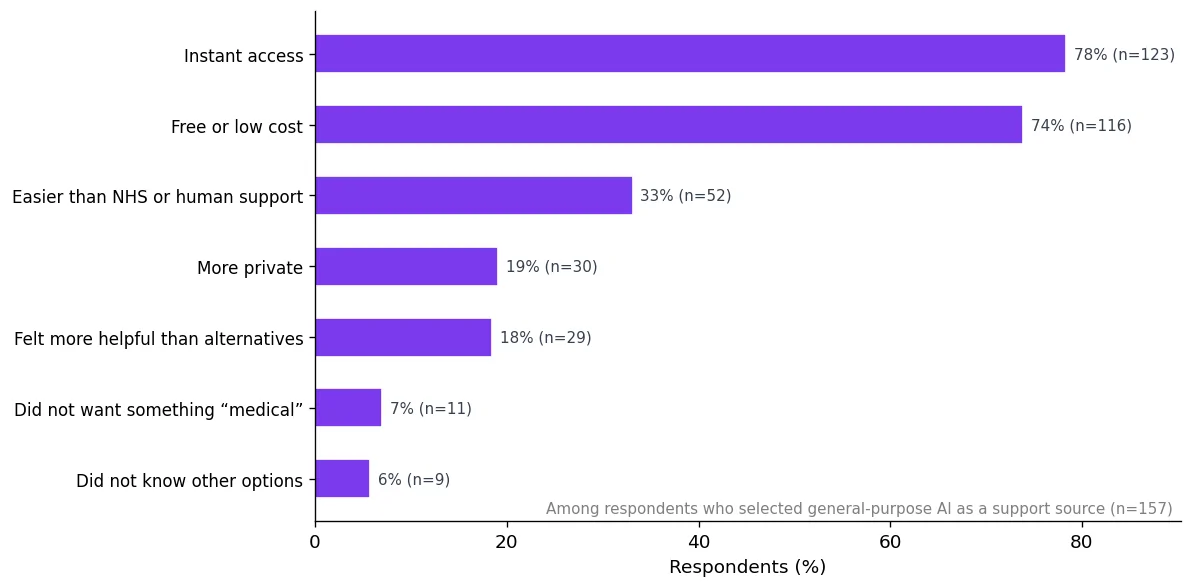
Motivations for choosing general-purpose AI tools for emotional support among respondents who endorsed general-purpose AI as a support source (n=157). Respondents could select up to 3 options. NHS: National Health Service.

### Perceived Change in Access to Support

Two items assessed perceived impact on well-being and access to support on an exploratory basis. Of the 364 who answered, 60.2% (219/364; 95% CI 55.1-65.1) reported that their access to mental health support had worsened since losing access to Ash, with 30.8% (112/364; 95% CI 26.2-35.7) reporting it was “much worse” and 29.4% (107/364; 95% CI 25.0-34.3) “a bit worse.” No change was reported by 31.6% (115/364; 95% CI 27.0-36.5), and 8.2% (30/364; 95% CI 5.8-11.5) reported improvement (a bit better: 4.7%, 17/364; 95% CI 2.9-7.4; much better: 3.6%, 13/364; 95% CI 2.1-6.0). The impact was most pronounced among the 26% (100/384; 95% CI 21.9-30.7) of respondents who had not sought any support since, 88 of whom answered this item: in this subgroup, 80.7% (71/88; 95% CI 71.2-87.6) reported worse access, including 56.8% (50/88; 95% CI 46.4-66.7) who rated it “much worse” and 23.9% (21/88; 95% CI 16.2-33.7) “a bit worse.” The remaining respondents in this subgroup reported no change (10.2%, 9/88; 95% CI 5.5-18.3) or improvement (9.1%, 8/88; 95% CI 4.7-16.9; a bit better: 2.3%, 2/88,; much better: 6.8%, 6/88).

### Current Well-Being

Among 363 who answered, 41.9% (152/363; 95% CI 36.9-47.0) reported that their general well-being over the past month had been worse than usual (slightly worse: 30.6%, 111/363; 95% CI 26.1-35.5; much worse: 11.3%, 41/363; 95% CI 8.4-15.0), while 25.9% (94/363; 95% CI 21.7-30.6) reported feeling better than usual (slightly better: 18.7%, 68/363; 95% CI 15.1-23.1; much better: 7.2%, 26/363; 95% CI 4.9-10.3) and 32.2% (117/363; 95% CI 27.6-37.2) reported no change. Among respondents who had not sought any support since losing access, the proportion reporting worse-than-usual well-being rose to 56.8% (50/88; 95% CI 46.4-66.7). In this subgroup, 37.5% (33/88; 95% CI 28.1-47.9) reported feeling slightly worse and 19.3% (17/88; 95% CI 12.4-28.8) much worse than usual, while 28.4% (25/88; 95% CI 20.0-38.6) reported no change and 14.8% (13/88; 95% CI 8.8-23.7) felt better than usual (slightly better: 12.5%, 11/88; much better: 2/88, 2.3%).

### Demand for Purpose-Built Well-Being AI

When asked whether they would use Ash again if it were available in the United Kingdom under a clear well-being framework, 89.3% (325/364) of those who answered (95% CI 85.7-92.1) said they were very likely (68.4%, 249/364; 95% CI 63.5-73.0) or somewhat likely (20.9%, 76/364; 95% CI 17.0-25.3) to do so. A total of 8.2% (30/364; 95% CI 5.8-11.5) said they were not sure, and 2.5% (9/364; 95% CI 1.3-4.6) said they were unlikely to return.

### Qualitative Findings

#### Overview

Of 272 free-text responses analyzed, 270 (99.3%) were coded to at least one substantive theme. The codebook comprised 5 domains encompassing 15 individual codes, with responses frequently spanning multiple themes (Table 3). Percentages reflect code frequencies and are not intended as population prevalence estimates.

**Table 3:** Content analysis of free-text responses (n=272). Codes are nonmutually exclusive; individual responses could be assigned to multiple themes. Percentages reflect the proportion of the 272 analyzable responses. Two (0.7%) responses were too ambiguous for reliable classification.

Theme		Values, n (%)			
**Evaluation of AI tools**					
	Inferior to Ash	62 (22.8)			
	Adequate or comparable	43 (15.8)			
	Critique of Ash	9 (3.3)			
**Support gap and disengagement**					
	No adequate replacement found				92 (33.8)
	Human support preferred but inaccessible				12 (4.4)
	NHS^a^ access barriers referenced				8 (2.9)
**Emotional response to loss**					
	Distress at losing access	43 (15.8)			
**Valued features of a purpose-built tool**					
	Insights and reflections valued			20 (7.4)	
	Memory and conversational continuity			19 (7)	
	Privacy, trust, and safety valued			9 (3.3)	
	24/7 on-demand availability valued			8 (2.9)	
**Coping strategies adopted**					
	Self-managing or informal coping		61 (22.4)		
	Switched to other well-being app		37 (13.6)		
	Accessing a human professional		36 (13.2)		
	VPN^b^ workaround to maintain access		8 (2.9)		

^a^NHS: National Health Service.

^b^VPN: virtual private network.

#### Evaluation of General-Purpose AI as Substitute

The most commonly discussed topic was users’ experience with general-purpose AI after losing Ash. A total of 22.8% (62/272) of coded responses described these tools as inferior, citing lack of conversational memory, generic rather than personalized responses, a tendency to validate rather than constructively challenge, and the absence of structured reflective features. As 1 respondent noted: “With ChatGPT it’s difficult because it doesn’t have memory and can’t ask ‘how did that thing go last week?’—it feels less personable and it does feel like AI compared to Ash.” A smaller group (15.8%, 43/272) found general-purpose AI comparable or acceptable: “ChatGPT. It works about the same. It seems to cut more to the point, which I like but can’t always remember the back story which is a draw back.” Several respondents (3.3%, 9/272) preferred the directive style of general-purpose AI, critiquing Ash’s repetitive conversational patterns and difficulty reaching a resolution: “Ash kept asking me questions non-stop and felt like there was never a resolution to things.”

#### Support Gap and Disengagement

Overall, 33.8% (92/272) of coded responses reported finding no suitable alternative to Ash. This was compounded by structural barriers: 4.4% (12/272) expressed a preference for human support but cited cost or inaccessibility, and 2.9% (8/272) specifically referenced NHS waiting times. Sample responses included the following: “There is nothing I can use. Other NHS-authorised apps do not cater for my needs” and “Bottling things up as I have no one to talk to.”

#### Emotional Response to Loss

A total of 15.8% (43/272) of coded responses expressed notable emotional distress at losing access to Ash, using language such as “gutted,” “devastated,” and “don’t know what I would do without it.”

#### Valued Features of a Purpose-Built Tool

Across several overlapping codes, respondents identified features that distinguished Ash from general-purpose alternatives: memory and conversational continuity across sessions (7% of coded responses, 19/272), structured insights and reflections (7.4%, 20/272), 24/7 on-demand availability (2.9%, 8/272), and the privacy and trust associated with a purpose-built, evidence-informed tool (3.3%, 9/272). While some of these features (eg, 24/7 availability and privacy) also characterize general-purpose AI, respondents emphasized that they found value in the combination of purpose-built design, conversational memory, and structured reflective features with Ash. In other words, the distinction was not accessibility alone, but the perceived safety and intentionality behind a purpose-built tool. One respondent captured several of these dimensions: “Ash’s killer feature was its weekly reflections. It has an amazing ability to see beyond the specifics of our conversations to the broader themes and challenges. Which is something Claude and ChatGPT don’t do that I’ve been missing a lot!” Another noted the trust dimension: “Other AI tools don’t feel safe due to news about suicides related to their usage. Ash was the only trustworthy app for me.”

#### Coping Strategies Adopted

Among coded responses, the most frequently coded theme overall was self-managing through informal means (22.4%, 61/272), including journaling, exercise, meditation, and leaning on friends or family. A total of 13.6% (37/272) participants described having switched to another well-being app, and 13.2% (36/272) participants were accessing a human professional—often noting it as a complement rather than a replacement. Furthermore, 2.9% (8/272) participants reported using a virtual private network to maintain access to Ash.

## Discussion

### Principal Findings

To our knowledge, this study provides the first empirical evidence of where users go when access to a purpose-built AI well-being tool is disrupted. For 53% (204/384) of respondents, the answer was general-purpose, unregulated AI (27%, 104/384), or nothing (26%, 100/384). Fewer reported seeking professional care from the NHS (5%, 19/384) or a private therapist (8%, 31/384) as their primary resource. These findings suggest that the self-reported use of formal, human-delivered mental health services may be relatively uncommon in the wake of access disruptions to purpose-built AI well-being tools; most former users of Ash reported turning to general-purpose alternatives or having a gap in care. Users who reported turning to general-purpose AI did so frequently but instrumentally, primarily for instant access and low cost rather than perceived superiority; most reported that their access to support had worsened and over 40% (152/363) reported worse-than-usual well-being; and in open-ended responses they generally characterized general-purpose AI as an inferior substitute for a purpose-built tool. These findings matter because AI regulation is evolving rapidly and carries real-world consequences: here, there was voluntary withdrawal in the context of regulatory uncertainty by the makers of a purpose-built model while leaving general-purpose models untouched, an asymmetry that our data suggest may carry measurable costs for the people it affects.

### Comparison With Prior Work

Prior work has documented the rising adoption of general-purpose AI for emotional support [2,3], evaluated the efficacy and safety of commercially available purpose-built tools [15,20-22,24], and assessed the capabilities and risks of general-purpose chatbots in controlled or observational settings [4,5]; none has examined the real-world impact of regulatory policy on help-seeking behavior among former users of a purpose-built AI for mental health. Our findings suggest that, in the wake of such policy, high usage of traditional health care is not observed; for most, there is reliance on the very general-purpose tools whose safety limitations this literature has documented [4-6,22], or no support at all. These patterns reflect an empirical instance of the disruption the NASSS framework locates outside the user-technology relationship [14], and of the “missing middle” left underserved [13].

Our quantitative and qualitative findings converge on a central point: the care usage reported by respondents appears more consistent with constrained choice than with preference, with motivations centering on access and cost rather than perceived superiority. Three mechanisms appeared to underlie this substitution: logistical barriers to formal care, psychological barriers rooted in stigma and prior negative experiences with services, and the specific design affordances of a purpose-built tool that general-purpose AI generally did not replicate. The dominant motivations for turning to general-purpose AI for mental health after losing access to Ash (instant access or cost-effectiveness) reflected logistical barriers to professional care, rather than enthusiasm for AI as a modality: nearly a third cited “easier than NHS or human support,” and fewer than 1 in 5 felt general-purpose AI was more helpful than alternatives. These reflect the choices of people navigating an access gap rather than exercising a preference. Users who tried ChatGPT or Claude as substitutes largely described them as lacking the qualities of a support relationship built with clinical intent: a system that remembered what they had shared across sessions, that surfaced patterns they could not see themselves, and that challenged their thinking rather than simply validating it. Users described how general-purpose AI could respond in the moment but often struggled to track a trajectory or push back. Notably, in open-text responses, the distinction respondents drew was not between AI and human support, but between a tool designed for psychological care and one repurposed for it.

It is also noteworthy that 3.3% (9/272) of free-text responses specifically raised trust and safety concerns about general-purpose AI, with some referencing news reports about suicides linked to chatbot use. This concern is particularly salient in a regulatory context where purpose-built tools face greater scrutiny than general-purpose alternatives. Recent research has documented a range of safety concerns with general-purpose AI for mental health: an analysis of real user interactions found that mental health crises were apparent in a nonnegligible minority of conversations, with companion AIs often unable to recognize or respond appropriately to signs of distress [4]. Emerging case reports have described phenomena including emotional dependency, reinforcement of delusional beliefs, and dangerous behavioral escalation [6]. A systematic review evaluating general-purpose generative AI chatbots for mental health found that while these tools show promise for psychoeducation and symptom monitoring, their diagnostic accuracy, cultural competence, and safety in crises remain limited [5]. These concerns especially apply to general-purpose platforms that may lack the crisis escalation protocols, boundary management, and safety architecture central to tools explicitly designed for well-being.

The 26% (100/384) of respondents who reported not having sought any support since losing access to Ash warrant particular attention. This group reported the worst outcomes across all measures: 81% (71/88) said their access to support had gotten worse (57%, 50/88 “much worse”), and 57% (50/88) reported worse-than-usual well-being over the past month. These individuals did not report using NHS services, did not find a private therapist, and did not turn to ChatGPT. They simply stopped seeking support. This population is likely invisible to policymakers and health systems: they do not appear in NHS referral statistics, are not captured by app marketplace data, and were not redirected to any alternative service. In their free-text responses, this subgroup of respondents described cost barriers, negative experiences with NHS services, and an unwillingness to start over with a tool they did not trust. The clinical significance of an abrupt loss of a trusted support tool for people managing everyday emotional difficulties, many of whom may lack other accessible options, deserves further investigation. While cost and access are important factors, our past research suggests that stigma and broken trust with human services also explain meaningful variance in AI help-seeking behavior [27].

These substitution patterns are consistent with the NASSS framework’s account of how regulatory withdrawal of a health technology cascades across domains [14], and with the “missing middle” literature documenting the persistent gap between primary and specialist mental health care [13]. While the present study did not assess clinical severity, the finding that so few users reported seeking professional care despite ongoing need suggests a population for whom viable alternatives simply did not exist.

### Limitations

Several limitations should be considered when interpreting these findings. First, the sample comprises former users of a specific product who were sufficiently engaged to respond to a survey and significantly more engaged than the average user; selection bias likely inflates positive sentiment toward Ash, particularly given the low response rate (5.3%, 393/7415). The 89.3% (325/364) intent-to-return figure, in particular, should be interpreted in this context. Second, all outcomes are self-reported and cross-sectional; we cannot establish causal relationships between service withdrawal and changes in well-being or access. For example, it is possible that users turned to tools such as general-purpose AI not because of service disruption, but rather because of factors such as preference and brand familiarity, insufficient differentiation of the purpose-built tool, or absence of clinical need among some users. Third, the single-item well-being measure lacks the psychometric properties of validated instruments and provides only a coarse assessment. Given these limitations, our findings from former users of a single product in a single country may not generalize to other tools or health systems. In addition, the qualitative coding was AI-assisted and refined through 3 rounds of human review but was not subjected to a formal intercoder reliability analysis. We also did not assess clinical severity and therefore cannot determine what proportion of respondents had needs warranting professional care. Finally, as the developer of the withdrawn product, Slingshot AI has a financial and reputational interest in these findings; we have attempted to mitigate this through transparent reporting of all results, including critiques of Ash and transparency around the subset of users who found general-purpose AI adequate.

Where possible, future research should follow users prospectively rather than cross-sectionally, to understand whether the access and well-being decrements observed here persist, worsen, or resolve. Incorporating validated clinical measures could help clarify how many displaced users had needs that warranted professional care, and comparison cohorts (eg, with users of other tools or users of the same tool in jurisdictions where access was uninterrupted) could help isolate the effect of service withdrawal itself. Replication beyond a single product and country is needed to establish how far these patterns generalize.

### Conclusion

Among the 5.3% (393/7415) of invited former users who responded to a survey after a purpose-built AI well-being tool was voluntarily removed from the UK market, general-purpose AI and no support were the 2 most frequently reported primary sources of care. These findings suggest that regulatory frameworks for AI well-being tools should account for the real-world care usage behavior observed in the wake of disrupted access to purpose-built products. They should also weigh a central asymmetry: the same regimes that constrain purpose-built tools typically leave the general-purpose AI platforms that users turn to next unregulated, so restricting the former may, paradoxically, increase exposure to the latter. Users are not waiting for regulatory clarity to seek emotional support through AI, and the alternatives they find may offer fewer safeguards than the tools they lost.

## Appendix

Multimedia Appendix 1 CHERRIES checklist.

Multimedia Appendix 2 Survey instrument.

Multimedia Appendix 3 Qualitative codebook.

## Abbreviations

BRANY: Biomedical Research Alliance of New York
CHERRIES: Checklist for Reporting Results of Internet E-Surveys
NASSS: Non-Adoption, Abandonment, Scale-up, Spread, and Sustainability
NHS: National Health Service
RQ: research question

## Data Availability

Deidentified quantitative data are available upon reasonable request. All code is publicly available on GitHub [28].
