# Supplementary material for: Support-Seeking After Market Withdrawal of a Purpose-Built AI Well-Being Tool (Ash): Cross-Sectional Survey
Source: JMIR Med Inform. 2026 Oct 13;14:e96515. doi: 10.2196/96515 (PMC13714522; doi:10.2196/96515)
Supplement: Supplementary file 1 [file medinform_v14i1e96515_app1.docx]

# CHERRIES Checklist

Completed Checklist for Reporting Results of Internet E-Surveys (CHERRIES; Eysenbach, J Med Internet Res 2004;6(3):e34).

| **Checklist item** | **How addressed in this study** |
| --- | --- |
| **Design** | |
| **Describe survey design** | Closed (invitation-only) cross-sectional web survey of former users of a purpose-built AI wellbeing application (Ash), conducted after its withdrawal from the UK market in January 2026. |
| **IRB approval and informed consent process** | |
| **IRB approval** | Reviewed by the BRANY SBER IRB (IRB00010793) and determined exempt under 45 CFR 46.104(d)(4)(ii). |
| **Informed consent** | Data were collected during routine product operations; users had consented at onboarding to the use of their data for research. No separate study-specific consent was taken (basis for the secondary-analysis exemption). Survey participation was voluntary. |
| **Data protection** | Analytic dataset de-identified prior to analysis; email addresses (collected for the optional prize draw) held separately and deleted after the draw. Processing complied with UK GDPR and the Data Protection Act 2018 (Article 6(1)(f) legitimate interests; Article 9(2)(j) research). |
| **Development and pre-testing** | |
| **Development and testing** | Items were developed by the research team to capture the specific constructs of interest. |
| **Recruitment process and description of the sample** | |
| **Open vs. closed survey** | Closed survey (invitation-only). |
| **Contact mode** | Email invitations sent to the registered email addresses of a random sample of eligible users drawn from the Ash user database. |
| **Advertising the survey** | Not publicly advertised; invitation-only. |
| **Survey administration** | |
| **Web/E-mail** | Web-based survey hosted on Tally (tally.so), distributed by email. |
| **Context** | Hosted on Tally; invitation links sent to eligible former Ash users. |
| **Mandatory/voluntary** | Voluntary; items could be skipped (no forced responses). |
| **Incentives** | Optional entry into a prize draw. |
| **Time/date** | Open February-March 2026. |
| **Randomization of items** | Items were presented in a fixed order (not randomized). |
| **Adaptive questioning** | N/A |
| **Number of items / pages** | 12 items / 4 pages |
| **Completeness check** | No pre-submission completeness check; items could be skipped, and partially completed responses were retained. |
| **Review step** | Yes; respondents could navigate back to review and change their answers before final submission. |
| **Response rates** | |
| **Unique site visitor / view rate** | 618 unique visits to the survey link were recorded in Tally analytics (8.3% of the 7,415 invited users opened the survey). |
| **Participation rate** | 63.6% (393 of the 618 who opened the survey responded). The overall invitation response rate was 5.3% (393/7,415 invited). |
| **Completion rate** | 92.6% (364 completed all items / 393 respondents). |
| **Preventing multiple entries from the same individual** | |
| **Cookies / IP / log / registration** | Each invited user received a unique, single-use survey link (Tally’s duplicate-submission prevention), limiting submissions to one per invitee. |
| **Analysis** | |
| **Handling of incomplete questionnaires** | All respondents included where they provided data; denominators vary by item and are reported. The 29 respondents who completed only the first half were included for the items they answered. |
| **Atypical timestamp** | No exclusions based on submission timestamp. |
| **Statistical correction** | None; unweighted descriptive analysis (proportions with 95% Wilson CIs). |
