# Supplementary material for: Support-Seeking After Market Withdrawal of a Purpose-Built AI Well-Being Tool (Ash): Cross-Sectional Survey
Source: JMIR Med Inform. 2026 Oct 13;14:e96515. doi: 10.2196/96515 (PMC13714522; doi:10.2196/96515)
Supplement: Supplementary file 2 [file medinform_v14i1e96515_app2.docx]

# Survey Instrument and Recruitment Email

A. **Survey Instrument**

The following items comprised the survey administered to former Ash users in the UK via Tally (tally.so) between February and March 2026. Response options are listed beneath each item. Participation was voluntary and items could be skipped; all respondents were shown all items (no adaptive branching), with “N/A” or “Never” options.

**Item 1 (multiple response). Since Ash/Slingshot stopped being available in the UK, where have you turned for emotional or mental health support? (Select all that apply.)**

- General-purpose AI tools (e.g. ChatGPT, Claude, Gemini, Copilot)
- Other mental health or wellbeing apps
- NHS services (GP, NHS Talking Therapies, etc.)
- Private therapist / counsellor
- Friends, family, or partner
- Support groups or community groups
- Workplace support / EAP
- Online forums or social media
- Books, podcasts, or self-help programs
- I haven’t sought support since
- Other (free text)

**Item 2 (single response). Which ONE of the above have you relied on most?**

- General-purpose AI tools (e.g. ChatGPT, Claude, Gemini, Copilot)
- Other mental health or wellbeing apps
- NHS services (GP, NHS Talking Therapies, etc.)
- Private therapist / counsellor
- Friends, family, or partner
- Support groups or community groups
- Workplace support / EAP
- Online forums or social media
- Books, podcasts, or self-help programs
- I haven’t sought support since
- Other (free text)

**Item 3 (single response). How often do you use general-purpose AI tools (such as ChatGPT, Claude, Gemini, or Copilot) for emotional or mental health support?**

- Daily
- A few times a week
- About once a week
- 1-3 times a month
- Less than once a month
- I’ve tried it once or twice
- Never

**Item 4 (single response). When you use general-purpose AI (such as ChatGPT, Claude, Gemini, or Copilot) for emotional support, how does it usually fit into your life?**

- It’s my main source of support
- It’s one of several sources I use
- It’s a backup when nothing else is available
- I use it occasionally or out of curiosity
- N/A
- Other (free text)

**Item 5 (multiple response; up to 3). What do you mainly use general-purpose AI for? (Select up to 3.)**

- Venting or feeling heard
- Advice or problem-solving
- Coping tools (e.g. grounding, reframing)
- Relationship or communication help
- Stress, anxiety, or sleep support
- Journaling or reflection prompts
- N/A
- Other (free text)

**Item 6 (multiple response; up to 3). Why did you choose a general-purpose AI tool (such as ChatGPT, Claude, Gemini, or Copilot) for emotional support? (Select up to 3.)**

- Free or low cost
- Instant access
- Felt more helpful than alternatives
- More private
- Easier than NHS or human support
- Didn’t want something “medical”
- Didn’t know other options
- N/A
- Other (free text)

**Item 7 (single response). Compared to when you had Ash, how has your access to support changed?**

- Much better
- A bit better
- About the same
- A bit worse
- Much worse

**Item 8 (single response). If Ash were available in the UK today under a clear wellbeing framework, how likely would you be to use it again?**

- Very likely
- Somewhat likely
- Not sure
- Somewhat unlikely
- Very unlikely

**Item 9 (single response). Thinking about my general wellbeing, over the past month I have felt…**

- Much better than usual
- Slightly better than usual
- The same as usual
- Slightly worse than usual
- Much worse than usual

**Item 10 (open-ended). Is there anything you’re using now that works better—or worse—than Ash did for you?**

- [Free-text response]

**Item 11 (single response). Age range**

- 18-24
- 25-34
- 35-44
- 45-54
- 55-64
- 65+
- Prefer not to say

**Item 12 (single response). UK region**

- England
- Scotland
- Wales
- Northern Ireland
- Prefer not to say

B. **Recruitment Email**

Hi there,

You previously used Ash in the UK, and we’d like to understand where you’ve turned for emotional or mental health support now that Ash is not available in the UK.

The survey takes about 5-7 minutes, is anonymous, and your responses will be used in aggregate to inform future product and policy work.

As a thank-you, you can optionally enter a free prize draw to win a £500 gift card* after completing the survey.

**[Take the survey]**

Please note that this is not a crisis service. If you need urgent support, please contact 999 or Samaritans (116 123).

Thank you for your time — we really appreciate your perspective.

**Open to UK residents ages 18+; one entry per person; no purchase necessary.*
