# Supplementary material for: Support-Seeking After Market Withdrawal of a Purpose-Built AI Well-Being Tool (Ash): Cross-Sectional Survey
Source: JMIR Med Inform. 2026 Oct 13;14:e96515. doi: 10.2196/96515 (PMC13714522; doi:10.2196/96515)
Supplement: Supplementary file 3 [file medinform_v14i1e96515_app3.docx]

Qualitative Codebook and Coding Procedures

**A. Overview of the coding procedure**

Free-text responses to the item “Is there anything you’re using now that works better—or worse—than Ash did for you?” were analyzed using qualitative content analysis according to this codebook. Of 351 free-text responses, 272 contained sufficient content for analysis (>5 characters, excluding single-word or uninformative entries).

Codebook development proceeded in two stages. First, the lead author read all responses and inductively identified recurring patterns, which were discussed and refined with the authorship team and organized into 15 non-mutually-exclusive codes across five domains. Second, each code was operationalized as a set of transparent keyword and phrase patterns (regular expressions). A large language model was used to assist in drafting candidate patterns from the code definitions; all patterns were reviewed, edited, and approved by the lead author.

Patterns were refined over three coding passes. After each pass, the lead author manually coded those that remained uncoded, also reviewing all responses to correct misclassifications, either by refining the shared patterns or by direct reclassification. The 24 direct reclassifications are summarized in Section D. Of the 272 responses, 270 (99%) were coded to at least one theme; the 2 responses that could not be reliably classified are characterized in Section E.

**B. Codebook**

***Domain 1. Evaluation of general-purpose AI as a substitute***

| **Code** | **Definition and decision rules** | **Anchor example** | **n (%)** |
| --- | --- | --- | --- |
| General-purpose AI inferior (T1) | Respondent evaluates general-purpose conversational AI (eg, ChatGPT, Claude, Gemini) as worse than Ash or inadequate for emotional support.  Include: Explicit unfavorable comparisons with Ash; general-purpose tools described as generic, impersonal, over-validating, or lacking memory; statements that no AI tool tried compares to Ash.  Exclude: Bare mention of using a general-purpose tool without evaluation (code T15); criticism directed at Ash itself (code T13). | *“With ChatGPT it’s difficult because it doesn’t have memory and can’t ask ‘how did that thing go last week?’—it feels less personable and it does feel like AI compared to Ash.”* | 62 (23%) |
| General-purpose AI adequate or preferred (T2) | General-purpose AI described as comparable to, an acceptable substitute for, or better than Ash.  Include: “Works about the same”; fills the gap; specific features preferred over Ash.  Exclude: Use without any evaluative content (code T15). | *“ChatGPT. It works about the same. It seems to cut more to the point, which I like but can’t always remember the back story which is a draw back.”* | 43 (16%) |
| Ash critique (T13) | Unfavorable evaluation of Ash itself.  Include: Repetitiveness, circular conversations, lack of resolution, missing features (eg, language support), technical problems specifically about Ash.  Exclude: Criticism of alternative tools (codes T1/T2). | *“Ash kept asking me questions non-stop and felt like there was never a resolution to things.”* | 9 (3%) |

***Domain 2. Support gap and disengagement***

| **Code** | **Definition and decision rules** | **Anchor example** | **n (%)** |
| --- | --- | --- | --- |
| No replacement found (T3) | No suitable alternative identified since service withdrawal.  Include: Explicit statements that nothing works or compares; short negative answers to the item prompt (eg, “No,” “Nothing really”); still searching for support.  Exclude: A named alternative in active use (codes T2, T6, T14). | *“There is nothing I can use. Other NHS-authorised apps do not cater for my needs.”* | 92 (34%) |
| NHS access barrier (T4) | References to NHS or primary care access barriers.  Include: Waiting lists or waiting times; unavailable GP, therapist, or mental health service appointments; overstretched services.  Exclude: Cost barriers to private care (code T5). | *“Ash was a great support NHS is awful to get help in my area.”* | 8 (3%) |
| Human support preferred but inaccessible (T5) | Stated preference for human support that is blocked by cost, availability, or access.  Include: Wanting a therapist or counsellor but unable to afford or access one; human support available but not accessible when needed.  Exclude: Currently receiving human professional support without an access concern (code T6). | *“A private therapist is better - but I would expect this! It's also more expensive…"* | 12 (4%) |

***Domain 3. Emotional response to loss***

| **Code** | **Definition and decision rules** | **Anchor example** | **n (%)** |
| --- | --- | --- | --- |
| Distress at loss of access (T11) | Emotional distress expressed about losing access to Ash.  Include: Grief or loss language (eg, “gutted,” “devastated”); missing Ash.  Exclude: Neutral preference statements without emotional content (codes T1/T3). | *“I miss using Ash and am gutted to be unable to use it.”* | 43 (16%) |

***Domain 4. Valued features of a purpose-built tool***

| **Code** | **Definition and decision rules** | **Anchor example** | **n (%)** |
| --- | --- | --- | --- |
| Memory and continuity (T7) | Conversational memory, continuity, personalization, or follow-up across sessions cited as a distinguishing feature that is valued in Ash or missed in alternatives.  Include: References to the tool remembering prior conversations, knowing the user’s history, or following up on earlier topics.  Exclude: Generic personalization claims with no reference to memory or continuity. | “The general AI tools aren't as good - they don't have a persistent memory and aren't trained on the same dataset that Ash is, it's obvious when you use them.” | 19 (7%) |
| Insights and reflections (T8) | Structured insights, reflections, or pattern identification cited as valued features.  Include: References to weekly reflections, summaries, or the tool surfacing patterns and themes across conversations.  Exclude: General praise without reference to reflective features. | *“Ash’s killer feature was its weekly reflections. It has an amazing ability to see beyond the specifics of our conversations to the broader themes and challenges.”* | 20 (7%) |
| 24/7 availability (T9) | On-demand, always-available, in-the-moment support cited as a valued feature.  Include: References to availability at any hour, immediacy, or support in the moment distress arises.  Exclude: Access framed purely as cost (code T5). | *“Talking with friends and family does help, but they aren’t available instantly, I don’t feel comfortable fully opening up and they aren’t trained.”* | 8 (3%) |
| Privacy, trust, and safety (T10) | Privacy, trust, safety, or non-judgment cited as valued features; comfort disclosing to AI relative to humans; safety concerns about alternatives.  Include: Non-judgmental support; things the respondent cannot share with people; distrust of general-purpose tools’ safety.  Exclude: Comments about seeing a private therapist. | *“Other AI tools don’t feel safe due to news about suicides related to their usage. Ash was the only trustworthy app for me.”* | 9 (3%) |

***Domain 5. Coping strategies adopted***

| **Code** | **Definition and decision rules** | **Anchor example** | **n (%)** |
| --- | --- | --- | --- |
| Human professional accessed (T6) | Currently accessing a human professional (therapist, counsellor, psychologist, GP, or reference to therapy such as CBT).  Include: Started or ongoing therapy; named professional support in current use.  Exclude: Preference for human support without current access (code T5). | *“Talking to certified therapist.”* | 36 (13%) |
| VPN workaround (T12) | Continued access to Ash via VPN or other workaround.  Include: Any reference to circumventing the withdrawal to keep using the service.  Exclude: N/A | *“No nothing compares to it. I have found ways around like using a VPN as there is really nothing else like it available.”* | 8 (3%) |
| Other wellbeing app (T14) | Use of another wellbeing app or digital platform, named or unnamed.  Include: Named apps (eg, Headspace, Wysa, GoalPOSTS); unnamed “another app” or “other resources.”  Exclude: General-purpose AI tools (codes T1/T2/T15). | *“GoalPOSTS works for me. It’s a practical framework that aligns well with my needs for process.”* | 37 (14%) |
| Self-management and informal support (T15) | Self-management or informal support as a current coping strategy.  Include: Journaling, exercise, meditation, faith, creative activities.  Exclude: Evaluative comparisons of AI tools (codes T1/T2). | *“Journaling without AI.”* | 61 (22%) |

**C. Illustrative classification patterns**

Each code comprised multiple case-insensitive regular expression patterns; a response received a code if any pattern matched. Full pattern lists (approximately 130 patterns across the 15 codes) are available in the analysis code. Two illustrative examples:

General-purpose AI inferior (T1): nothing.{0,20}(compares?|comes? close|works? as well|like ash|as good) — matches statements such as “nothing compares to Ash” or “nothing comes close.”

24/7 availability (T9): (24.?7|always available|always there|any ?time|middle of.{0,10}night|3.?am|whenever) — matches references to round-the-clock or in-the-moment availability.

**D. Manual reclassifications (n=24)**

Twenty-four responses were reclassified by the lead author where classification patterns did not match but the content clearly fit a theme. These were predominantly short or implicitly worded responses: statements implying no replacement had been found without stating so directly (n=8, coded “No replacement found”), mentions of informal coping resources without evaluative content (n=5, “Self-management and informal support”), implicit favorable comparisons of general-purpose AI (n=3, “General-purpose AI adequate”), and references to valued design features (n=2 each for “Memory and continuity” and “Insights and reflections”, with one response receiving both). One response each was reclassified to “General-purpose AI inferior,” “Human professional accessed,” “Privacy, trust, and safety,” “Ash critique,” and “Other wellbeing app.” Each reclassification is documented individually, with the response identifier and rationale, in the study’s records.

**E. Responses not classifiable (n=2)**

Two responses lacked sufficient specificity for reliable classification and were excluded from theme counts: one was a generic statement about wellness support with no codable referent, and one was a brief response mentioning communication, without sufficient context to assign a code.
